# An *in vitro* model to study molecular pathogenesis of sarcopenia established by a SASP-dependent human myotube culture

**DOI:** 10.1371/journal.pone.0326968

**Published:** 2025-07-07

**Authors:** Kiyo-aki Ishii, Ryo Hashimoto, Chikako Umeda, Tohru Hosoyama, Ken Watanabe

**Affiliations:** 1 Department of Musculoskeletal Disease, National Center for Geriatrics and Gerontology (NCGG), Obu, Japan; 2 Department of Biochemistry, Graduate School of Medical Sciences, Nagoya City University, Nagoya, Japan; 3 Biobank, NCGG, Obu, Japan; Fujita Health University: Fujita Ika Daigaku, JAPAN

## Abstract

Sarcopenia is a condition that affects one’s activities of daily livingand is rapidly increasing with the ages of the global population. However, the basic molecular mechanisms for prevention and treatment are not fully understood. Although rodent model animals have many valuable aspects for studying sarcopenia, some aspects and mechanisms differ from humans, such as immune response, metabolism, stress response, and myofiber composition. This study established a human cell-based *in vitro* model to elucidate the molecular mechanism by which SASP from senescence-induced human mesenchymal stem cells led to the narrowing of human myotube diameter, suggesting that this model is useful for studying sarcopenia. Gene expression profiling was performed the molecular mechanisms and devel on the model by RNA sequencing to identify genes whose expression was affected by SASP. Among these, the exposure to SASP upregulated *PDK4* expression, and a PDK4 inhibitor, DCA, could increase myotube diameter and reverse SASP-mediated narrowing of the diameter. Pathway analyses suggested that SASP affected energy metabolism by activating OXPHOS and promoting the expression of mitochondrial function-related genes and mitochondrial biosynthesis factors. These results provide insights that contribute to developing new treatments for sarcopenia.

## Introduction

Sarcopenia is an age-related condition diagnosed based on a decrease in muscle mass and quality, with a reduction in physical function being a sign of severe sarcopenia [1,2]. Sarcopenia is significantly associated with risk for falls, fractures, hospitalization, and death. This emphasizes the need for screening and early diagnosis of sarcopenia [3]. To improve diagnostic accuracy and establish prevention and treatment methods, it is essential to elucidate the pathogenesis, including the molecular mechanisms, but this has not yet been achieved [4,5].

In young mice intraperitoneally transplanted with senescent cells, skeletal muscle-related activities such as the maximum walking speed, grip strength, and hanging endurance decreased dose-dependently after one month, and the effect was sustained for 6 months. Whereas the transplanted senescent cells originated from the adipose tissue, those increased the expression of senescence-related genes in the muscles, implying that a small number of transplanted non-muscle senescent cells can cause long-term, systemic senescence, especially in skeletal muscles [6].

Skeletal muscle is composed of multinucleated skeletal muscle cells called myofibers and a population of mitotic mononuclear cells, such as satellite cells, fibro-adipose progenitor cells (FAPs)/Mesenchymal progenitors (MPs), endothelial cells, pericytes, macrophages, neurons, tendon cells, and others, that exist in the interstitial microenvironment and are necessary for homeostasis and adaptation [7]. Besides senescence of the myofibers per se, senescence of the adjacent interstitial cells may also affect the myofibers by secreting the senescence-associated secretory phenotype (SASP), including inflammatory cytokines, chemokines, and proteases, resulting in sarcopenia [8].

FAP/MP is an interstitial population of mesenchymal stromal cells that can differentiate into fat, bone, and cartilage and play an essential role in muscle homeostasis, repair, and regeneration [9]. With age, the function of FAP/MP declines, and the myogenic potential of muscle stem cells (MuSCs) is suppressed [10]. Rat myoblasts co-cultured with senescent MPs lost their ability to form myotubes and reduced the expression of fusion proteins in the myoblasts, suggesting a mechanism by which senescent MPs inhibit myoblast fusion [11]. When 22-month-old female rats were subjected to resistance training, the number of senescence FAPs/MPs and SASP factors was reduced compared to the control group [12].

However, because there are significant differences between humans and rodents in important physiological aspects such as immune response, metabolism, stress response, and myofiber composition (ratio of fast and slow muscles), the results from studies in rodents may not seem to be directly applicable to sarcopenia in humans. It is also proposed that muscular atrophy in the disuse model shows a different pathological state from sarcopenia [13].

In this study, a senescent cell model system was constructed using human MSCs and human skeletal muscle cells to investigate the effects of SASP released from other tissue cells on skeletal muscle. Myotubes were collected explicitly from this model system, and pathway analysis was performed through gene expression profiling.

## Materials and methods

### Cell culture

Human Mesenchymal Stem Cells (MSCs) from Takara Bio were cultured with Mesenchymal Stem Cell Growth Medium 2 (Takara Bio) in early passages and then cultured to be acclimated in 4.5g/L high glucose Dulbecco modified Eagle medium (DMEM; Gibco) containing 10% fetal bovine serum (FBS; JRH Biosciences) and 1% penicillin/streptomycin (FUJIFILM Wako Pure Chemical Corporation). Induction of the senescence of the MSCs was essentially according to d-Sen protocol [14]. After growing to 80–90% confluence, cells were treated with 100 nM doxorubicin hydrochloride (FUJIFILM Wako Pure Chemical Corporation). After 24 hours, the medium was replaced with DMEM containing 10% FBS and 1% penicillin/streptomycin, and the medium was replaced every two days. After 2 weeks, the culture supernatant was collected, filtered through a 0.45 μm polyethersulfone filter (Millex, Millipore), and used as the SASP-containing medium.

Human myogenic cell clone Hu5 KD3 (Hu5) [15] were cultured in DMEM containing 20% FBS, 2% Ultroser G (PALL Life Science) and 1% penicillin/streptomycin ( growth medium; GM) at 37ºC (10% CO₂) on collagen-coated six well dishes (Sumilon, SUMITOMO BAKELITE). After growing up to confluency, replace the medium with DMEM containing 2% horse serum (Gibco), 1% Insulin-Transferrin-Selenium (Gibco), and 1% penicillin/streptomycin (differentiation medium; DM). The culture medium was changed every two days, and the cells were differentiated after culturing for one week.

### γH2AX and Hoechst staining

Immunofluorescence staining was performed using the DNA Damage Detection Kit - γH2AX - Green (Dojindo). The culture medium was removed from the cell culture plates, and the plates were fixed at room temperature for 5 minutes with 250 mM HEPES buffer containing 4% paraformaldehyde and 0.1% Triton X-100. After fixation, the supernatant was removed, and the cells were washed twice with PBS. Next, PBS containing 1% Triton X-100 was added, and the cells were incubated at room temperature for 20 minutes to allow penetration. After washing twice with PBS, the blocking solution was added, and the cells were incubated at room temperature for 20 minutes. After blocking, wash twice with PBS, add the γH2AX antibody fixative solution to the kit, and incubate at room temperature for 1 hour. Then, wash twice with PBS, add the secondary antibody staining solution, and incubate again at room temperature for 1 hour. After staining, wash twice with PBS, add Hoechst 33258 solution (Dojindo) to achieve a final concentration of 10 μg/ml, and incubate at 37°C for 30 minutes. Finally, wash twice with PBS and acquire fluorescent images using an IX71 fluorescence microscope (Olympus) and Cell Sens imaging software (Olympus). Fluorescence intensity was measured using a SpectraMax Paradigm microplate reader (Molecular Devices).

### Myotube diameter measurement.

After Hu5 cell differentiation, control, 10 ng/ml IL-1β (Peprotech), 1 mM or 5 mM DCA (FUJIFILM Wako Pure Chemical Corporation), SASP, IL-1β + DCA, and SASP+DCA were added to the myotubes, respectively, and after 3 days, they were photographed using IX71 (Olympus). Cell Sens imaging software (Olympus) was used to measure the diameter. Six wells (5 locations/well) were measured per reagent.

### Myotube collection

TrypLE Express Enzyme (Gibco) was added to Hu5 cells after differentiation, and the cells were placed at 37°C (5% CO₂) for 10 minutes. The cells peeled off the dish were separated into undifferentiated cells and myotubes using a 100 μm strainer (Falcon). The strainer containing the myotubes was inverted, and the myotubes were placed in a 50 ml tube using PBS. The myotubes were collected by centrifugation at 1000 rpm for 5 minutes (Fig 3A).

**Fig 1:**
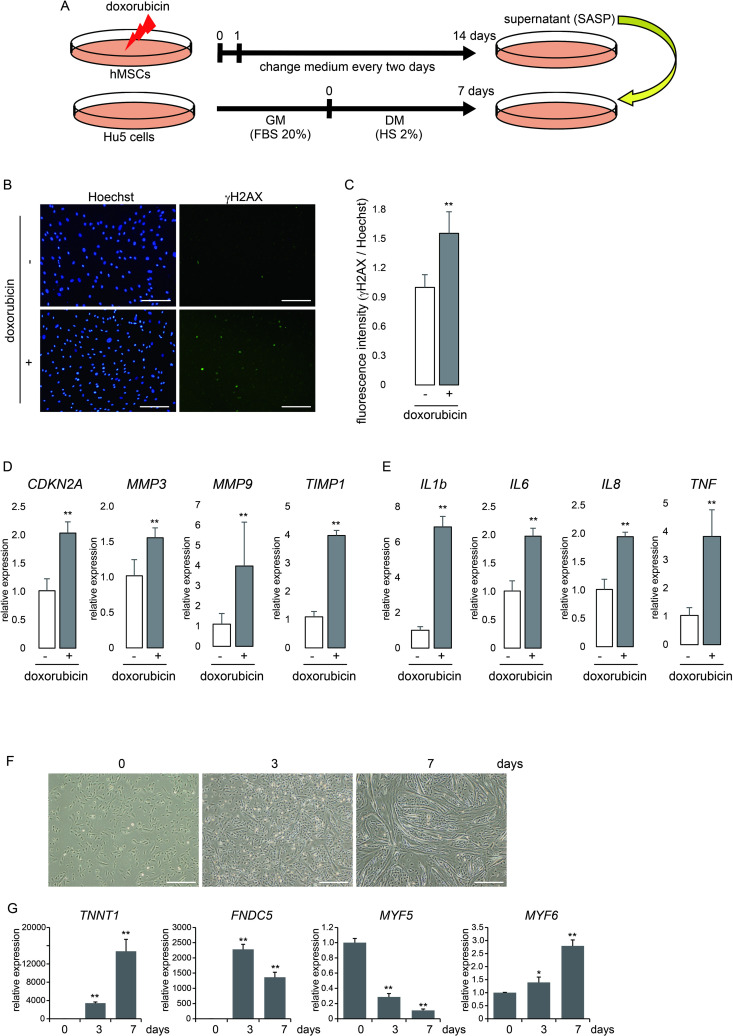
SASP-induced human skeletal muscle senescent cell model and the effects of DCA. (A) Human myotube senescent model. To induce the senescence of human MSCs, the cells were treated with doxorubicin for 24 hours and then removed. After 14 days, the conditioned medium of the d-Sen MSC culture was added to human myotubes differentiated from myoblasts (Hu5) for 7 days. (B) A representative Hoechst and γH2AX staining images of human MSCs treated with or without doxorubicin (scale bar, 250 μm). (C) Quantification of fluorescence intensity (γH2AX/Hoechst). (D) Gene expression of senescence-related markers by real-time PCR. Culture of human MSCs with or without doxorubicin. **p < 0.01. (E) Gene expression of SASP markers by real-time PCR. Culture of human MSCs with or without doxorubicin. **p < 0.01. (F) Representative image of Hu5 cells differentiation. (scale bar, 500 μm). (G) Gene expression of muscle differentiation markers by real-time PCR. The days indicate as duration of culturing of Hu5 cells with DM. *p < 0.05, **p < 0.01. Statistical analyses were conducted using the student’s *t*-test.

### Quantitative real-time PCR

Total RNA was extracted from cultured Hu5 cells using RNeasy Plus Universal (Qiagen) according to manufacturer’s protocol. According to the manufacturer’s protocol, cDNA was synthesized from 100 ng of total RNA using a SuperScript IV VILO Master Mix (Invitrogen). Quantitative real-time PCR was performed using TB Green Premix Ex Taq II (Takara bio) and the CFX96 Touch (Bio-Rad). Target gene expression in each sample was normalized to express the endogenous control gene (*RPS18*).

### RNA sequencing

According to the manufacturer’s protocol, total RNA was isolated from the Hu5 culture using RNeasy Plus Universal. RNA quality and quantity were estimated using NanoVue (GE HealthCare). The RNA sequencing was done in Macrogen, Japan. cDNA was synthesized by random hexamer primers (TruSeq stranded mRNA LT Sample Prep Kit; Illumina). TruSeq was performed using the NovaSeq 6000 S4 Reagent Kit v1.5 on NovaSeq 6000 Version 1.7.0 (Illumina).

**Fig 2:**
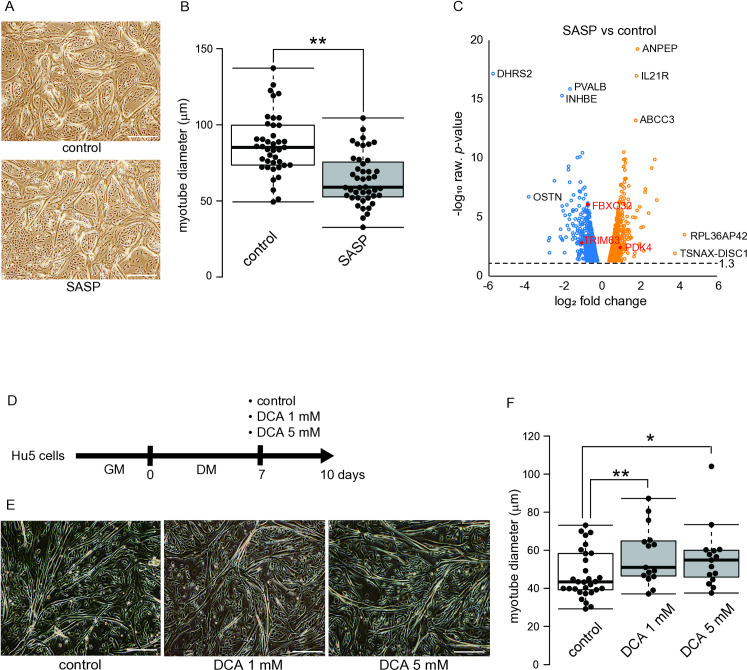
Effects of SASP and DCA on differentiated Hu5 cells. (A) A representative image of Hu5 myotubes treated with or without SASP. upper; control, lower; SASP, (scale bar, 500 μm). (B) Myotube diameter (μm), **p < 0.01. (C) Volcano plot of gene expression changes between the SASP and control groups. Blue and orange dots represent downregulation and upregulation (*p* < 0.05), respectively. (D) After induction of the myotubes (7 days in DM), the myotubes were exposed to media containing DCA (1 mM or 5 mM) for 72 hours. (E) The representative images of myotube culture 3 days after exposure to DCA. left; control, center; DCA 1 mM, right; DCA 5 mM (scale bar, 500 μm). (F) Myotube diameter (μm), *p < 0.05, **p < 0.01. hMSCs, human mesenchymal stem cells; Hu5, human myoblast cell line Hu5 KD3; GM, growth medium, DM, differentiation medium. Statistical analyses were conducted using the student’s *t*-test.

The differentially expressed genes identified by RNA-seq analysis are provided in S1 Dataset, including gene symbols, log2 fold changes, and p-values. These data were used for generating volcano plots (Figs. 2C, 4D) and Ingenuity Pathway Analysis (Figs. 5–7).

**Fig 3:**
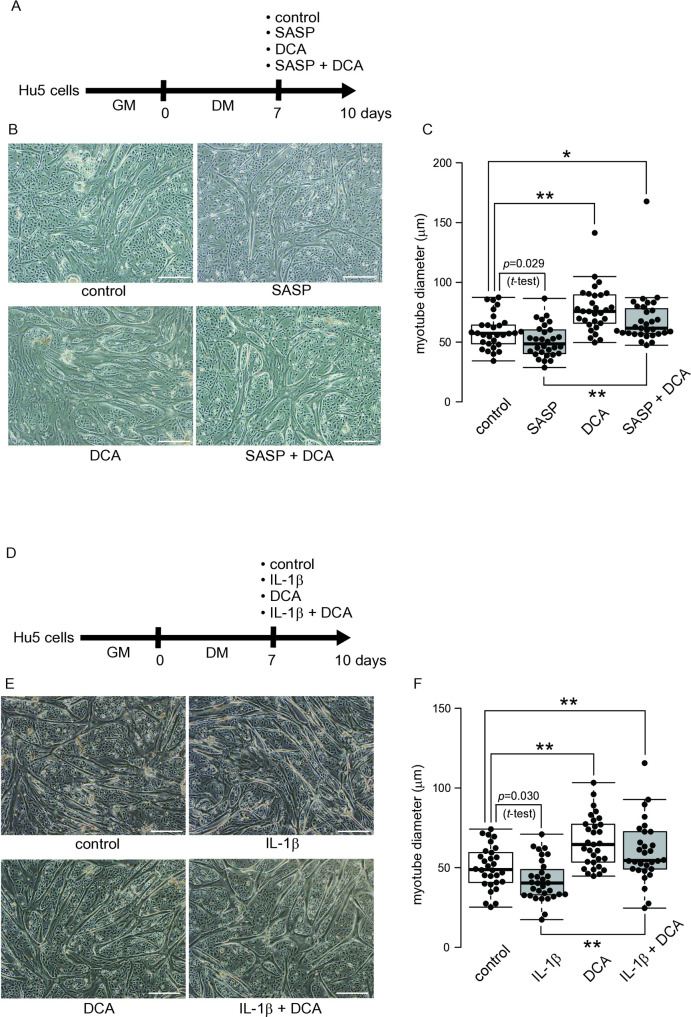
Effects of DCA on SASP and IL-1β responses in differentiated Hu5 Cells. (A) After induction of the myotubes (7 days in DM), the myotubes were exposed to media containing either DCA (1 mM), SASP medium, or DCA (1 mM) + SASP medium, respectively. (B) The representative images of the myotube cultures 3 days after exposures as (A). scale bar, 500 μm. (C) Myotube diameter (μm), ^#^*p* < 0.05, *p < 0.05, **p < 0.01. (D) After induction of the myotubes (7 days in DM), the myotubes were exposed to media containing either DCA (1 mM), IL-1β (10 ng/ml), or DCA (1 mM) + IL-1β (10 ng/ml), respectively. (E) The representative images of the myotube cultures 3 days after exposures as (D). scale bar, 500 μm. (F) Myotube diameter (μm), *p < 0.05, **p < 0.01. Statistical analyses were conducted using the Student’s *t*-test and one-way ANOVA *post hoc*.

**Fig 4:**
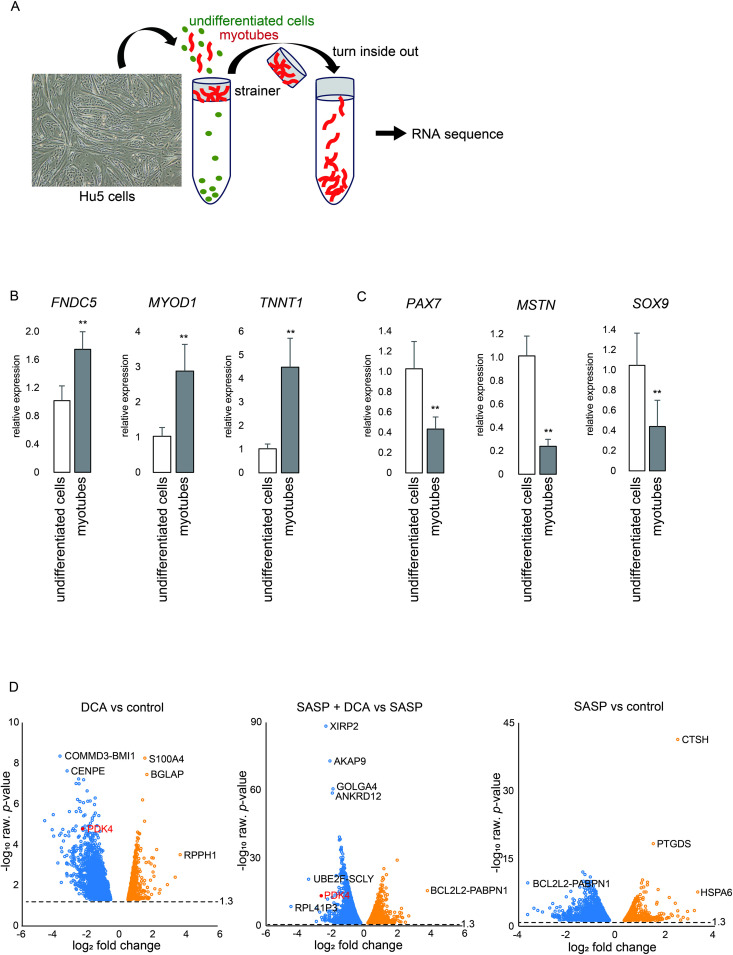
Effects of DCA and SASP on myotubes. (A) Separation of myotubes by a strainer (100 μm mesh) from the cultures. (B) Gene expression of differentiation markers by real-time PCR. **p < 0.01. (C) Gene expression of undifferentiated cell markers by real-time PCR. **p < 0.01. (D) Volcano plot of gene expression changes between the DCA and control groups (left), SASP+DCA and SASP groups (center), SASP and control groups (right), respectively. Blue and orange dots represent downregulation and upregulation (p < 0.05), respectively. Statistical analyses were conducted using the Student’s *t*-test.

**Fig 5:**
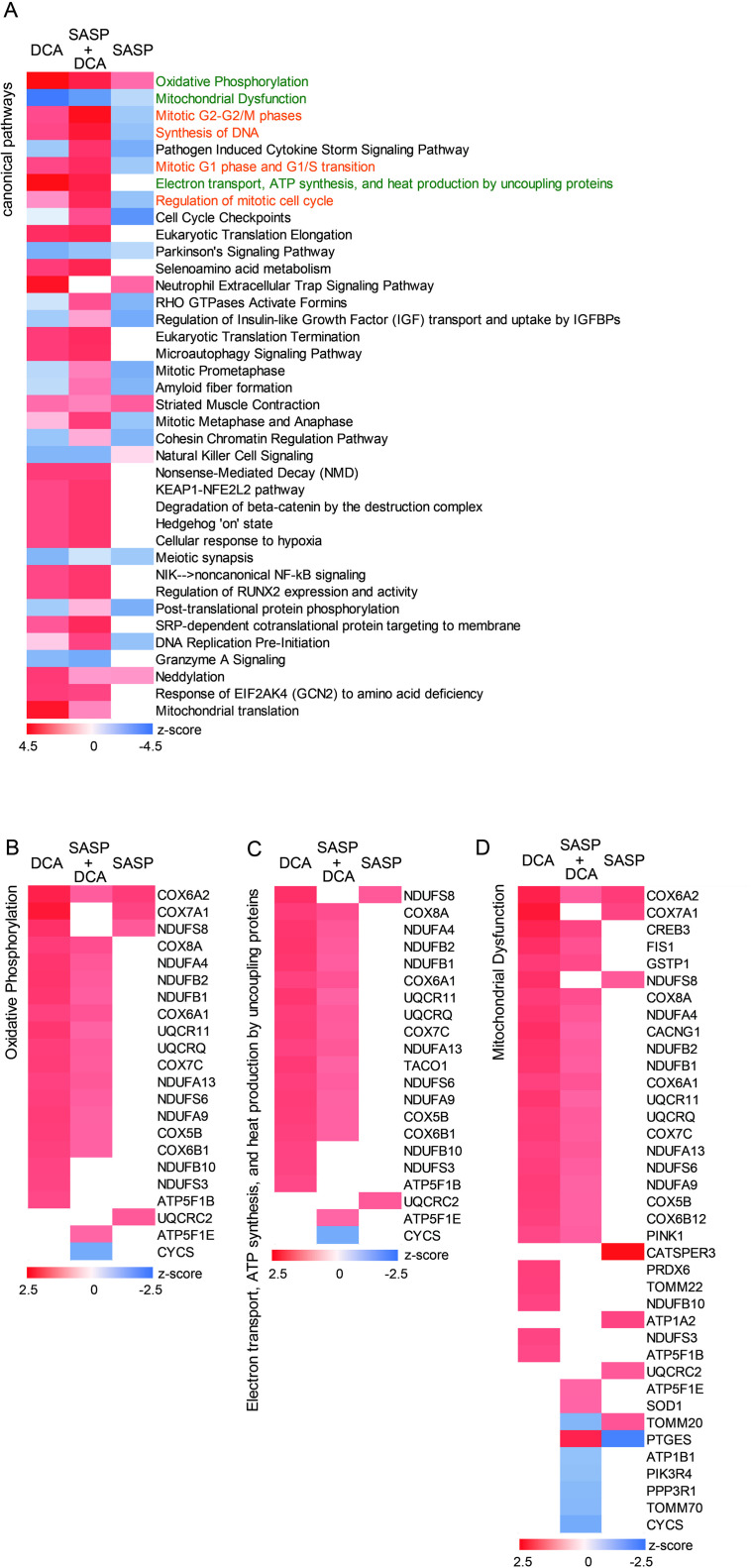
DCA activates the pathway of mitochondrial respiratory chain and its functions. (A) Heatmap comparing mRNA expression by IPA canonical pathways analysis of the myotubes treated with DCA (1 mM), SASP+DCA (1 mM), or SASP. Heatmap analysis of genes present in each pathway as follows: (B) Oxidative Phosphorylation; (C) Electron transport, ATP synthesis, and heat production by uncoupling proteins; (D) Mitochondrial Dysfunction.

**Fig 6:**
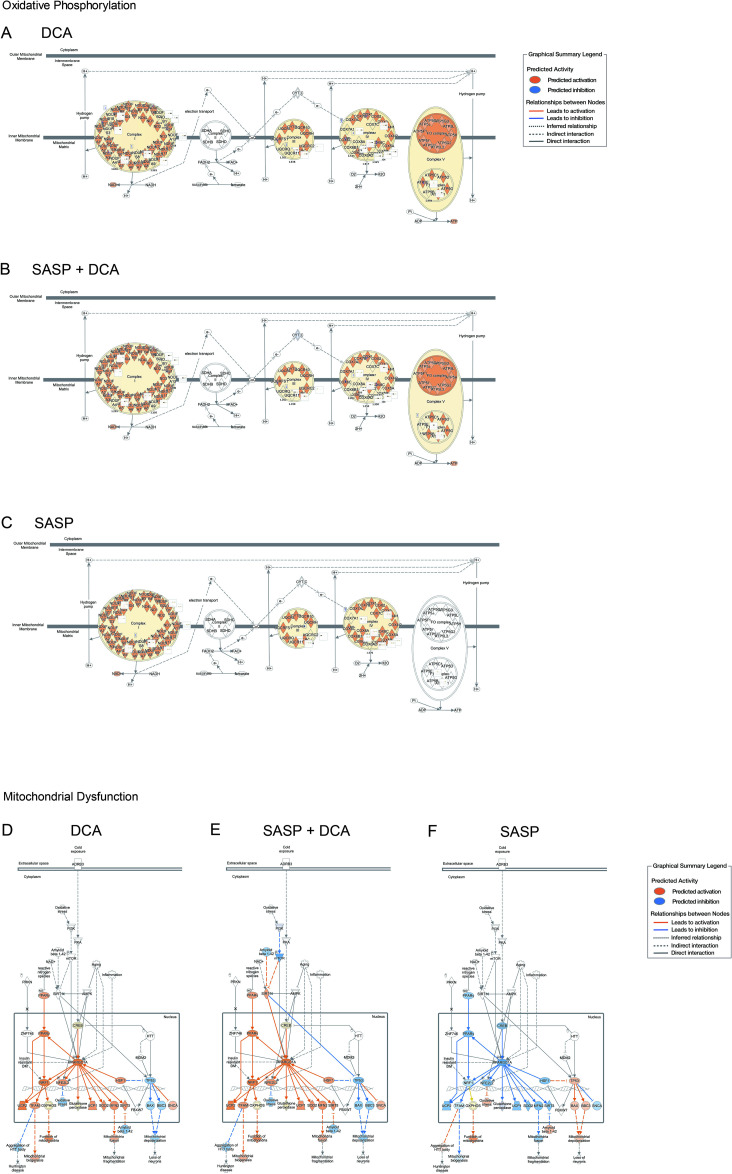
DCA activates the network of mitochondrial respiratory chain and its functions. IPA gene interaction network analysis of oxidative phosphorylation, (A) DCA, (B) SASP+DCA, (C) SASP. IPA gene interaction network analysis of Mitochondrial Dysfunction, (D) DCA, (E) SASP+DCA, (F) SASP.

**Fig 7:**
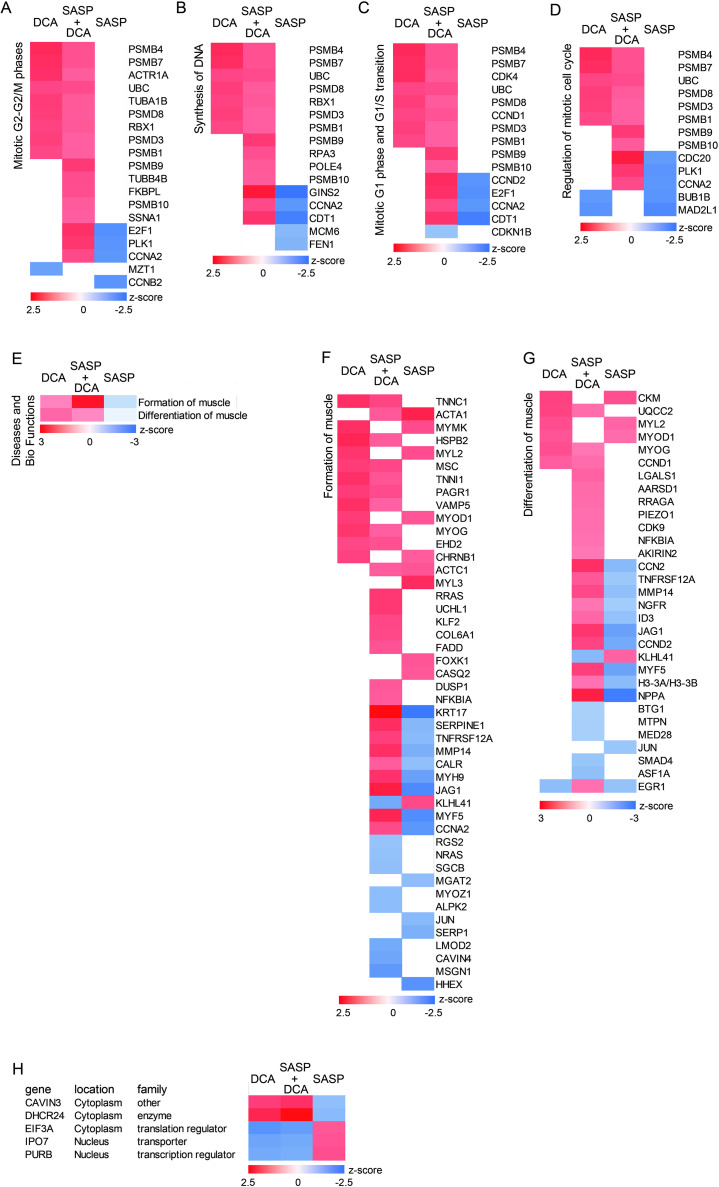
DCA activates the pathways of cell cycles and muscle function. Heatmap analyses of genes present in each pathway as follows: (A) Mitotic G2-G2/M phases; (B) Synthesis of DNA; (C) Mitotic G1 phase and G1/S transition; (D) Regulation of mitotic cell cycle. (E) Heatmap analysis of Diseases and BioFunctions. (F) Formation of muscle; (G) Differentiation of muscle. (H) Heatmap of the genes exhibited the expression patterns corresponding to the changes in the myotube diameter listed with the subcellular localization of the proteins encoded by the genes.

### Statistical analysis

Data are expressed as the mean ± s.d. Statistical analyses were conducted using the Student’s *t*-test and one-way ANOVA *post hoc*. Values were considered statistically significant at *p* < 0.05.

## Results

### Construction of a SASP-induced human skeletal muscle senescent cell model

To investigate the effects of SASP derived from MSCs on skeletal muscle, the SASP-induced human skeletal muscle senescence model was constructed using an immortalized human myoblast cell line, Hu5 (Fig 1A). To induce senescence in MSCs, doxorubicin was added to the culture medium of human MSCs for 24 hours to induce SASP secretion [14], evidenced by the marked staining with γH2AX, a DNA damage marker and one of the indicators of cellular senescence. As shown in Fig 1B, C, a higher number of γH2AX-positive cells were detected in the senescence-induced human MSCs. The expression of senescence-related genes such as *CDKN2A*, *MMP3*, *MMP9*, and *TIMP1* was upregulated in the senescence-induced cells (Fig 1D). Furthermore, analysis of the expression of representative SASP genes showed the significant increase in the expression of *IL1B*, *IL6*, *IL8*, and *TNF* in the senescence-induced MSCs (Fig 1E). Thus, it can be considered that the collected supernatant contains at least these representative SASP factors. This culture supernatant was added to human myotubes differentiated from myoblasts. These human myotubes underwent muscle differentiation for 7 days, resulting in increased expression of muscle differentiation marker genes TNNT1, FNDC5, and MYF6, while expression of the myoblast marker gene MYF5 decreased (Fig 1F, G). In addition, the diameter of the myotubes was narrower than that of the control group (Fig 2A, B). Myotube narrowing often indicates muscle senescence and sarcopenia [16–18]. Therefore, we applied this SASP-induced *in vitro* muscle senescence model to human myotubes and investigated sarcopenia-related genes and molecular mechanisms.

### Human myotubes are thickened by treatment with dichloroacetic acid (DCA), an inhibitor for pyruvate dehydrogenase kinase 4 (PDK4)

RNA sequencing was performed to profile the gene expression and compare skeletal muscle cells from the SASP-added and control groups. The genes that were specifically upregulated (log_10_ raw. *p*-value > 13) were alanyl aminopeptidase, membrane (*ANPEP*) (log_2_ FC = 1.80), interleukin 21 receptor (*IL21R*) (log_2_ FC = 1.75), and ATP binding cassette subfamily C member 3 (*ABCC3*) (log_2_ FC = 1.71). The genes encoding dehydrogenase/reductase 2 (*DHRS2*) (log_2_ FC = −5.82), parvalbumin (*PVALB*) (log_2_ FC = −1.76), and inhibin subunit beta E (*INHBE*) (log_2_ FC = −2.18) were down-regulated (-log_10_ raw. *p*-value > 15) specifically by SASP treatment. On the other hand, the expression of *FBXO32* and *TRIM63*, encoding atrogin-1 and MuRF1, respectively, was reduced by exposure to SASP (Fig 2C).

The present study centered on the augmentation of PDK4 gene expression engendered by SASP. PDK4, a protein that impedes the transformation of pyruvate to acetyl-CoA using its inhibitory phosphorylation of the pyruvate dehydrogenase complex, has recently been documented to exhibit elevated expression in biopsy specimens of lower limb muscles from elderly subjects (mean age 75 years) in comparison to those from young subjects (mean age 20 years) [19]. DCA, an inhibitor of PDK4, was added to the differentiated myotube cell culture medium (Fig 2D), and the myotube diameter was measured. As a result, adding DCA led to an increase in myotubes diameter (Fig 2E, F).

### DCA improved SASP factor-induced myotube narrowing

After confirming the effect of DCA on increasing myotube diameter, the effect of DCA on myotube thinning caused by the SASP factor was determined. SASP, DCA, or both were added to the differentiated myotube culture (Fig 3A). SASP decreased the myotube diameter. Still, DCA increased it, as shown in Fig. 2F. Simultaneous addition of SASP and DCA suppressed the decrease in myotube diameter caused by the exposure to SASP alone, and the myotube diameter was increased more than that of the control (Fig 3B & C). In particular, IL-1β, a major component of the SASP factor [20], was added to the myotube culture. IL-1β and DCA were also administered simultaneously (Fig 3D). Similar to the exposure to SASP, the treatment with IL-1β reduced the diameter of the myotubes. The simultaneous treatment of IL-1β and DCA not only reversed the IL-1β-dependent decrease in myotube diameter, but DCA increased the diameters more than in the control (Fig 3E & F).

### SASP factors inhibit the oxidative phosphorylation (OXPHOS) in human myotubes

The culture of myotubes generated from myoblasts in the differentiation medium still contains not only myotubes but also a considerable number of undifferentiated cells (Fig 1F). To remove unfused, undifferentiated cells, the cultured cells were separated by passing through a cell strainer to collect myotubes (Fig 4A). To confirm the efficient separation, the profiles of cells that passed through the cell strainer (undifferentiated cells) and cells present on the cell strainer (myotubes) were characterized, the expression of the differentiation marker genes *FNDC5*, *MYOD1*, and *TNNT1* was significantly higher in the cells present on the strainer (myotubes) than the cells in the flow through (Fig 4B). In contrast, the expression of the undifferentiated cell marker genes *PAX7*, *MSTN*, and *SOX9* was substantially high in the cells that passed through the cell strainer (undifferentiated cells) (Fig 4C). The gene expression profiling of the collected myotubes were conducted by RNA sequencing after exposure to each SASP, DCA, and SASP and DCA simultaneously. When comparing DCA and control and simultaneous exposure to SASP and DCA with exposure to SASP alone, it is evident that DCA induced suppression of PDK4 gene expression in both conditions (Fig 4D).

To elucidate the molecular mechanism involved in changes in myotube diameter by treatment with SASP or DCA, pathway analysis was performed using Ingenuity Pathway Analysis (IPA) software. By Ingenuity Canonical Pathways analysis, the activation and inhibition in functional pathways were extracted and listed according to the Z-score for each SASP, DCA, and SASP+DCA. The top of the list included the pathways with genes related to the mitochondrial respiratory chain complex. “Oxidative Phosphorylation” and “Electron transport, ATP synthesis, and heat production by uncoupling proteins” were activated by DCA, while “Mitochondrial Dysfunction” was suppressed by DCA (Fig 5A-D). By focusing the gene expression associated with each mitochondrial function, it was shown that the expression of genes about complex I (NADH ubiquinone oxidoreductase, NDUF), complex III (ubiquinol-cytochrome c reductase, UQCR), and complex IV (cytochrome c oxidase, COX) was augmented by DCA. Interestingly, SASP specifically suppressed the expression of genes related to complex IV (ATP synthase). Additionally, DCA did not activate only complex II (succinate dehydrogenase) of the respiratory chain complex (Fig 6A-C). Moreover, *PPARGC1A*, a master regulator of mitochondrial biogenesis, was activated by DCA and inhibited by SASP. Concomitantly, DCA increased the expression of its downstream genes, such as *UCP1*, *SOD2*, and *MFN2*, which had been inhibited by SASP (Fig 6D-F).

### The narrowing of myotubes caused by the SASP factor is related to ubiquitin-proteasome activity related to the cell cycle

The IPA canonical pathways analysis showed that SASP also inactivates the cell cycle in addition to mitochondrial function. The results were “Mitotic G2-G2/M phases”, “Synthesis of DNA,” “Mitotic G1 phase and G1/S transition”, and “Regulation of mitotic cell cycle” (Fig 5A). After completing the Canonical Pathways analysis, in which the genes involved in the cell cycle/DNA synthesis were identified, an investigation was conducted into the genes associated with the ubiquitin-proteasome system. It was ascertained that many genes belonging to the ubiquitin-proteasome system, particularly those encoding 20s proteasomes, were implicated (Fig 7A-D).

### SASP disturbed gene expression of the pathways in muscle homeostasis

The IPA Diseases & Functions analysis was applied to examine the biological ontology of gene expression for DCA, SASP+DCA, and SASP treatment, respectively. As a result, it was found that “formation of muscle” and “differentiation of muscle” have been established in this human model experimental system (Fig 7E). As focusing on the individual genes within each pathway, it was observed that those genes central to muscle contraction, including *TNNC1* and *TNNI1*, the myogenic regulatory factors (MRFs) *MYOG* and *MYF5*, and the downstream MRF-regulated genes *CCND1*, *VAMP5*, and *EHD2*, which control myoblast fusion, and *UQCC2*, a mitochondrial nucleoid-like protein that regulates ATP production, were listed (Fig 7F, G).

On the other hand, the genes that exhibit characteristic expression patterns in response to the action of SASP or DCA, *i.e.*, potential candidates as the biomarkers, were examined with a focus on their protein localization. Although neither secreted nor plasma membrane proteins were extracted, genes *CAVIN3*, *DHCR24*, *EIF3A*, *IPO7*, and *PURB*, encoding proteins localized in the cytoplasm and/or nucleus, were detected (Fig 7H).

## Discussion

*In vitro* culture models have been adopted in rat L6, mouse C2C12 cell lines, or primary muscle cells collected from humans or animals. The factors such as oxidative stress (H_2_O_2_), phosphosphingolipids (ceramide or palmitic acid), inflammatory cytokines (TNF-α), and dexamethasone have been directly applied to the myotubes to understand the molecular mechanisms involved in the development and the intervention of sarcopenia [21]. However, in recent years, it has been proposed that senescent cells accumulate in the tissues as they age and induce senescence in the surrounding normal cells through the “bystander effect,” causing age-related diseases and functional decline, including sarcopenia [16]. In this study, senescence-induced human MSCs were used as the source of SASP, and an *in vitro* model was constructed in which the secreted SASP was applied to human myotubes to mimic myofiber deflation in sarcopenia. The SASP-induced human myotube narrowing was accompanied by characteristic gene expression changes. *In vitro*, myotube narrowing is often utilized as a surrogate indicator to model muscle senescence and sarcopenia-related atrophic changes, reflecting morphological features comparable to those observed in myofibers *in vivo* [22,23].

Although the expression of *FBXO32* and *TRIM63*, so-called atrogenes, is known to be upregulated through activation of the FOXO transcription factor in many cases of muscle atrophy [24], our results showed that the expression was downregulated by exposure to SASP. It has been reported that the expression of both genes is even reduced in the skeletal muscles of aging rats and mice with sarcopenia [25,26], and suggested that sarcopenia may be developed through different pathways from the relatively acute, FOXO-mediated muscular atrophy. Of note, no consistent results in the expression of *FBXO32* and *TRIM63*, rather not increased, have been obtained in studies of human sarcopenia [27], suggesting that sarcopenia may be developed through different pathways from the relatively acute muscular atrophy.

Expression of *PDK4* is upregulated in the skeletal muscles of the old compared to the young people [19]. Similarly, in rodents, PDK4 gene expression has also been reported to be promoted in cases of cancer cachexia [28], LPS antibiotics (metronidazole) [29], and muscle atrophy due to amyotrophic lateral sclerosis (ALS) [30,31]. A PDK4 inhibitor, DCA, not only increased myotubes diameter on its own but also showed resistance to the diameter reduction of the myotubes induced by SASP or IL-1β. It has been reported that DCA administration has beneficial effects in mice and rats, including improving the effects of endurance training in diabetic mice [32], delaying the onset of symptoms of ALS [30], and inhibiting statin myopathy [33]. DCA has been proposed as a possible therapeutic that is administered to patients with genetic mitochondrial diseases as a deficiency in PDH complex [34].

Gene expression profiling and pathway analyses have suggested that the oxidative phosphorylation pathway of mitochondria was suppressed by SASP and activated by DCA. The phenomenon of down-regulation of the electron transport chain and oxidative phosphorylation pathway in mitochondria with senescence has been observed in humans and other animals such as mice, rats, and rhesus monkeys [35,36]. In particular, the decrease in oxidative phosphorylation complex is more pronounced in monkeys and humans. ATP production in mitochondria was reduced in the skeletal muscles of elderly humans [35,37]. These findings are consistent with the present analysis of the oxidative phosphorylation pathway, in which the treatment with SASP markedly reduced the expression of genes related to complex V (ATP synthase), demonstrating the validity of the present human model system. In addition, our analysis showed that the gene expression of complex II (succinate dehydrogenase, *Sdh*) was unaffected. Notably, although the muscle-specific Sdh KO mice exhibited a decrease in mitochondrial oxygen consumption, myotube contractility, and exercise endurance, any changes in muscle mass, myotube type composition, or whole-body composition have been observed in the mice [38]. This may be related to the finding that the level of citrate synthase activity was not altered in young and elderly skeletal muscle biopsies [35]. In the future, it will be necessary to clarify how changes in the activity of individual complexes, rather than the overall activity, in the oxidative phosphorylation pathway affect skeletal muscle.

The pathway analysis also showed a prediction of activated mitochondrial function. The primary gene for mitochondrial function is *PPARGC1A*, and the expression levels of downstream genes were also increased by DCA treatment but decreased by exposure to SASP, similar to those of *PPARGC1A*. It has been reported that the expression levels of *PPARGC1A*, as well as its downstream target genes was decreased in skeletal muscle of the elderlies and that mRNA levels were decreased in various atrophic states, such as denervation, reduced loading, and type 2 diabetes mellitus [39]. Whereas a study has also reported that *PPARGC1A* expression was increased in skeletal muscle treated with DCA [40], the detailed mechanism is still unknown. Neither our pathway analyses predicted the upstream effector of the *PPARGC1A* gene expression.

Although this study suggests that PDK4 is involved in the molecular mechanism of sarcopenia-like myotube thinning caused by SASP, it remains to be clarified how PDK4 causes mitochondrial dysfunction and its relationship with SASP factors. PDK4 inhibits pyruvate dehydrogenase in the mitochondria by phosphorylation, thereby lowering the flow of carbon from the glycolytic pathway to the TCA cycle and shifting energy metabolism to a state where lipid oxidation predominates. This shift causes a decrease in ATP production efficiency and an increase in mitochondrial ROS production, accumulating oxidative stress in muscle cells and promoting mitochondrial dysfunction [31]. A link between the overexpression of PDK4 and mitochondrial dysfunction in the skeletal muscles of aged mice has been shown, and it has been reported that PDK4 causes changes such as a reduction in mitochondrial membrane potential, a decrease in the expression of oxidative phosphorylation complexes, and a decrease in ATP-generating capacity [31]. Furthermore, about how PDK4 is affected by SASP factors, single nuclei profiling has observed an increase in the expression of SASP factors (such as IL-6 and IL-1β) and co-expression of PDK4 in aged muscle cells, suggesting that SASP may act as an inducer of PDK4 [19]. In addition, it has been reported that in the cancer-related senescence microenvironment, SASP factors induce PDK4 expression in tumor stromal cells and thereby metabolic restructuring promotes lactate production [41], suggesting that SASP activates PDK4.

Another pathway, cell cycle, was ranked in the extracted canonical pathways. It has been thought that during muscle regeneration the DNA synthesis/cell cycle is activated in the muscle stem cells intrinsic to myofibers, and proliferation and differentiation begin [42–44]. However, it has recently been shown that when myofibers are damaged, the muscle nuclei move to the injured area, promoting transcription and translation [45]. Furthermore, it has been described that in myofibers under mechanical stress, DNA synthesis/cell cycle promotion occurs in the muscle nuclei independently of muscle stem cells [46–48]. In fact, the pathway related to DNA synthesis and the cell cycle was more active in the SASP+DCA group, where myotube regained the thickness, than in the DCA alone group. Whereas the relationship between the cell cycle and the proteasome has been investigated [49], the mechanism of how DCA regulates the expression of these proteasome-related genes is still unclear. The expression of genes related to the 20s proteasome, such as proteasome 20S subunit beta (*PSMB*)*1*, *4*, *7*, *9*, and *10*, was increased by DCA treatment. The 20S proteasome executes ubiquitin-independent protein degradation, and in addition to degrading proteins as a whole, it also cleaves at specific sites of the target proteins to generate functional degradation products. This affects various cellular functions, including the cell cycle, transcription, translation, and stress response [50]. The 20S proteasome gene expression by the treatment with DCA and SASP+DCA may also have affected the cell cycle and differentiation of MuSCs. As shown by the pathway analysis, the SASP inhibited the formation of muscle and differentiation of muscle pathways.

SASP may contribute to muscle senescence cells by suppressing the IGF signaling pathway. *IGFBP4*, *IGFBP5*, and *IGFBP7* are expressed in senescence cells, suppressing the IGF-1 pathway and inhibiting muscle regeneration [51–53]. IGFBPs are also associated with senescence pathways and have been suggested to influence SASP signaling [54], suggesting a role in muscle atrophy through IGF regulation and senescence related mechanisms. In our pathway analysis, “IGF transport and uptake by IGFBPs” was also extracted at a high rank, following mitochondrial metabolism and cell cycle-related pathways, suggesting the involvement of the IGFBP family (Fig 5A).

In fact, we attempted to use this system to search for genetic markers of SASP-induced myotube narrowing or recovery by DCA, but failed to identify any candidates for the specific markers that would be secreted extracellularly and may be detected in body fluids. Besides that, condition-specific expression of some genes, of which products are present intracellularly, was identified as follows: caveolae-associated gene *CAVIN3* [55], the subunit gene EIF3A [56] of translation initiation factor 3 (eIF3), and the nuclear-cytoplasmic transport receptor gene *IPO7* [57,58]. The lipid metabolism-related gene DHCR24 has been suggested to play an important role in maintaining the circadian clock in muscles [59]. Purine-rich element binding protein B encoded by *PURB*, which was upregulated by exposure to SASP, has been reported to inhibit the proliferation and differentiation of myoblasts in collaboration with circular RNA [60], implying a possibility that the target genes, but so far unknown, of the transcriptional regulator, PURB, might include the candidates for secreted proteins as the skeletal muscle-derived markers.

The screening strategy this time was to use transcriptome analysis (RNA-seq) to identify the genes whose expression was altered by SASP and then to extract candidates that might be detected in body fluids as secreted proteins. However, because the number of candidate genes with known secretory signal peptides or annotations related to extracellular transport was not significantly reduced, it was stated that specific extracellular biomarker candidates could not be identified at this stage. This situation may be due to the diversity and cell-type specificity of SASP. Basisty et al. analyzed the SASP proteome of multiple cell types and reported that the composition of SASP varies greatly depending on the cell type and stimulation conditions [61]. However, Perez et al. have shown that candidate markers derived from SASP are involved in muscle senescence and sarcopenia by analyzing the muscle tissue transcriptome and serum proteome parallel in aged muscle [19], justifying our proteomic follow-up strategy based on transcriptomic data. Therefore, although the RNA-seq data from this study did not lead to the direct detection of secreted proteins, it is a powerful starting point for future proteome analysis. Recently, Fernández-Lázaro et al. proposed 13 candidate extracellular biomarkers related to sarcopenia using proteomics and proteogenomics [62], and it will be necessary to analyze conditioned medium using a similar proteomic approach in the future.

In this study, an *in vitro* model was constructed to analyze the effects of SASP on skeletal muscle, thereby gaining insight into the molecular mechanisms and the treatment of sarcopenia. The SASP secreted by senescence-induced human MSCs upregulated *PDK4* expression, along with the thinning of myotubes derived from human myoblast Hu5. DCA reversed the reduced myotube diameter, probably through the promotion of cell differentiation by activating OXPHOS and improving mitochondrial function and energy metabolism. Although sarcopenia is multifactorial and the results from human subjects are considered heterogeneous, clarification of each pathological aspect and the situation is deemed necessary for understanding the pathogenesis and developing diagnostic and therapeutic seeds. This human myotube model is expected to reproduce at least some elements of sarcopenia pathology, and it is anticipated that the molecular analysis will be applied to diagnosis and treatment, including patient and pathological stratification. The human model may also be potentially helpful in screening anti-sarcopenia drugs.

## Supporting information

S1_Dataset Differentially expressed genes from RNA-seq analysis. This dataset includes gene symbols, log2 fold changes, and p-values used for RNA-seq-based gene expression profiling.
(XLSX)
